# Micellization: A new principle in the formation of biomolecular condensates

**DOI:** 10.3389/fmolb.2022.974772

**Published:** 2022-08-29

**Authors:** Tomohiro Yamazaki, Tetsuya Yamamoto, Tetsuro Hirose

**Affiliations:** ^1^ Graduate School of Frontier Biosciences, Osaka University, Suita, Japan; ^2^ Institute for Chemical Reaction Design and Discovery, Hokkaido University, Sapporo, Japan; ^3^ Institute for Open and Transdisciplinary Research Initiatives (OTRI), Osaka University, Suita, Japan

**Keywords:** long non-coding RNA (lncRNA), architectural RNA (arcRNA), micellization, block copolymer (BCP), NEAT1, paraspeckle, biomolecular condensate, phase separation

## Abstract

Phase separation is a fundamental mechanism for compartmentalization in cells and leads to the formation of biomolecular condensates, generally containing various RNA molecules. RNAs are biomolecules that can serve as suitable scaffolds for biomolecular condensates and determine their forms and functions. Many studies have focused on biomolecular condensates formed by liquid-liquid phase separation (LLPS), one type of intracellular phase separation mechanism. We recently identified that paraspeckle nuclear bodies use an intracellular phase separation mechanism called micellization of block copolymers in their formation. The paraspeckles are scaffolded by NEAT1_2 long non-coding RNAs (lncRNAs) and their partner RNA-binding proteins (NEAT1_2 RNA-protein complexes [RNPs]). The NEAT1_2 RNPs act as block copolymers and the paraspeckles assemble through micellization. In LLPS, condensates grow without bound as long as components are available and typically have spherical shapes to minimize surface tension. In contrast, the size, shape, and internal morphology of the condensates are more strictly controlled in micellization. Here, we discuss the potential importance and future perspectives of micellization of block copolymers of RNPs in cells, including the construction of designer condensates with optimal internal organization, shape, and size according to design guidelines of block copolymers.

## Introduction

Intracellular phase separation, which induces the formation of biomolecular condensates, is a fundamental mechanism of cellular compartmentalization. Such condensates play essential functions, including reaction crucible, sequestration, and chromatin hubs (Banani et al., 2017; Shin and Brangwynne, 2017; Alberti et al., 2019; Lyon et al., 2020; Sabari et al., 2020). RNA molecules are ubiquitously present in most condensates and play critical roles in the formation and function of biomolecular condensates (Yamazaki et al., 2019; Roden and Gladfelter, 2020). RNAs contribute to the formation of biomolecular condensates by increasing molecular interactions, such as RNA-RNA and RNA-protein interactions (Roden and Gladfelter, 2020; Yamazaki and Hirose, 2021), whereas excessive amounts of RNAs nonspecifically interacting with prion-like RNA-binding proteins (RBPs) inhibit phase separation of the RBPs (Maharana et al., 2018). Intermolecular interactions between RNA and protein molecules, including RNA-RNA, RNA-protein, and protein-protein interactions, contribute to the formation of biomolecular condensates by phase separation (Van Treeck and Parker, 2018).

Liquid-liquid phase separation (LLPS) is well studied as an intracellular phase separation mechanism and is a phenomenon with which the system is separated into domains of liquid phases with different molecular compositions, analogous to the case of salad dressing that separate into an oil-rich phase and a water-rich phase (Shin and Brangwynne, 2017). LLPS is driven by the multivalent interactions between prion-like or low complexity domains, oligomerization domains, and modular interacting domains (e.g., RNA binding domains) (Sanders et al., 2020; Banani et al., 2022). Strong molecular interactions, such as hydrogen bonding, electrostatic interactions between multivalent ions, and hydrophobic interactions, can act as transient crosslinks (Tanaka, 2011). With such strong molecular interactions, proteins in biomolecular condensates can transiently assemble networks that make the condensates viscoelastic (Mittag and Pappu, 2022).

Another important class of phase separation is microphase separation, with which the system forms multiple stable condensates (microphases) in the sea of different composition (Leibler, 1980; Semenov, 1985; Ohta and Kawasaki, 1986; Fredrickson and Helfand, 1987; Matsen and Schick, 1994; Matsen and Bates, 1996; Bates and Fredrickson, 1999; Safran, 2003). Meanwhile, we have recently identified that paraspeckle nuclear bodies scaffolded by RNAs and RBPs use micellization, which is distinct from LLPS and will be described in the following sections, in their formation in living cells (Yamazaki et al., 2021). Micellization has been studied for many decades, but for *in vitro* systems of amphiphiles (Tanford, 1972; Tanford, 1974a; Tanford, 1974b), synthetic polymers (Halperin and Alexander, 1989; Zhulina et al., 2005; Mai and Eisenberg, 2012; Moughton et al., 2012; Bates and Bates, 2017), and several polypeptides/proteins (Dreher et al., 2008; Williamson et al., 2010; Crick et al., 2013; McDaniel et al., 2014; Warner et al., 2017; Newcombe et al., 2018; Posey et al., 2018; Cable et al., 2019; Rana et al., 2021) at the thermodynamic equilibrium. Paraspeckle nuclear bodies are different from these systems because they are scaffolded by RNA-protein complexes (RNPs) and are seeded by transcription that drives the system out of the equilibrium, as described in the following sections (Yamazaki et al., 2021).

In this article, we first describe the roles of RNAs as scaffolds for biomolecular condensates and discuss why these molecules are suitable for such a purpose. We then describe the micellization of RNPs and discuss why cells use micellization to construct biomolecular condensates.

## RNAs as scaffolds of biomolecular condensates

**FIGURE 1:**
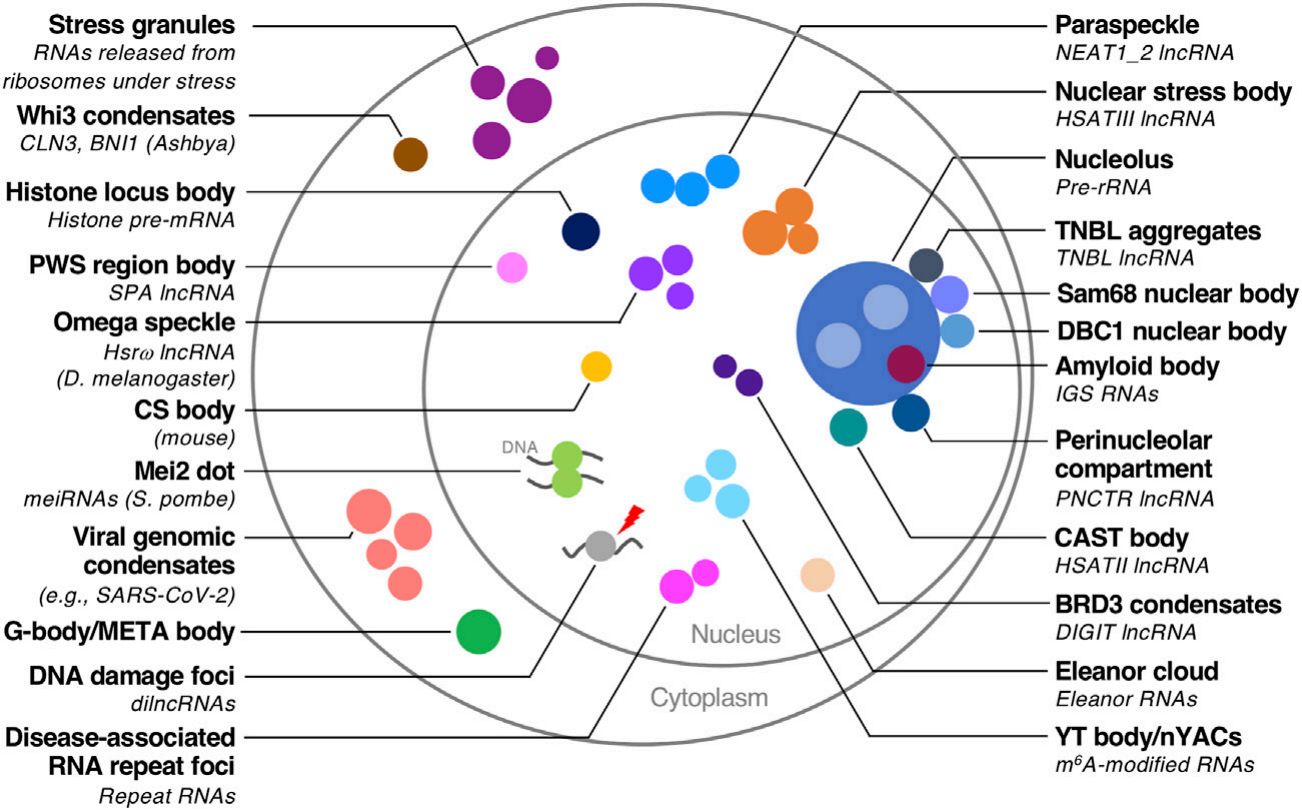
Biomolecular condensates with RNA scaffolds. Biomolecular condensates with RNA scaffolds in various organisms are illustrated. The scaffold RNAs are described in parentheses if they are identified. Species other than humans are also described in parentheses.

A class of RNAs termed architectural RNAs (arcRNAs) can be essential scaffolds of biomolecular condensates (Chujo et al., 2016; Chujo and Hirose, 2017; Yamazaki, 2018, Yamazaki et al., 2019). The arcRNAs are localized and enriched in the specific biomolecular condensates, and the removal of the RNA disrupts the condensates (Chujo et al., 2016; Chujo and Hirose, 2017). Dozens of cellular condensates are scaffolded by arcRNAs, which are found among various organisms ranging from yeast to humans (Nakagawa et al., 2021; Yamazaki et al., 2019; Yap et al., 2018; Miura et al., 2013; Fuller et al., 2020; Mannen et al., 2016; Mannen et al., 2021; Abdalla et al., 2019; Ninomiya et al., 2019; Ninomiya et al., 2021; Biamonti and Vourc’h, 2010; Shevtsov and Dundr, 2011; Hall et al., 2017; Wu et al., 2016; Audas et al., 2016; Daneshvar et al., 2020; Dumbovic et al., 2018; Lee et al., 2021; Cheng et al., 2021; Yamashita et al., 1998; Ding et al., 2019; Hiraoka, 2020; Michelini et al., 2017; Iserman et al., 2020; Cubuk et al., 2021; Savastano et al., 2020; Lu et al., 2021; Zhang et al., 2015; Langdon and Gladfelter, 2018; Sharp et al., 2022; Sabari et al., 2020; Hernandez-Verdun, 2011; Berry et al., 2015; Feric et al., 2016; Lafontaine et al., 2021; Prasanth et al., 2000; Ishizuka et al., 2014; Decker et al., 2022) (Figure 1). Pathologically expanded, repeat-containing RNAs are also regarded as a type of arcRNA (Wojciechowska and Krzyzosiak, 2011; Swinnen et al., 2019; Ninomiya and Hirose, 2020) (Figure 1). The arcRNA list was expanded by several recent studies, suggesting that phase separation is a widely used mode of action in RNA functions, including many long non-coding RNAs (lncRNAs) (Yamazaki et al., 2019; Chujo et al., 2017; Yap et al., 2018; Quinodoz et al., 2021; Elguindy and Mendell, 2021).

Transcription as a seeding event (nucleation) initiates the formation of biomolecular condensates constructed by nuclear arcRNAs near the transcription site (Mao et al., 2011; Shevtsov and Dundr, 2011). The produced RNAs recruit RBPs that possess domains prone to self-assembly, such as low complexity and coiled-coil domains. These molecular interactions induce phase separation by increasing the local concentration of these RBPs (Yamazaki et al., 2018; Yap et al., 2018; Quinodoz et al., 2021). Our recent work with the soft matter physics theory describes the mechanism and dynamics of such a phase separation driven by transcription of arcRNAs and inhibited by degradation of arcRNAs (Yamamoto et al., 2020a).

## RNAs are suitable biomolecules for scaffolds of biomolecular condensates

**FIGURE 2:**
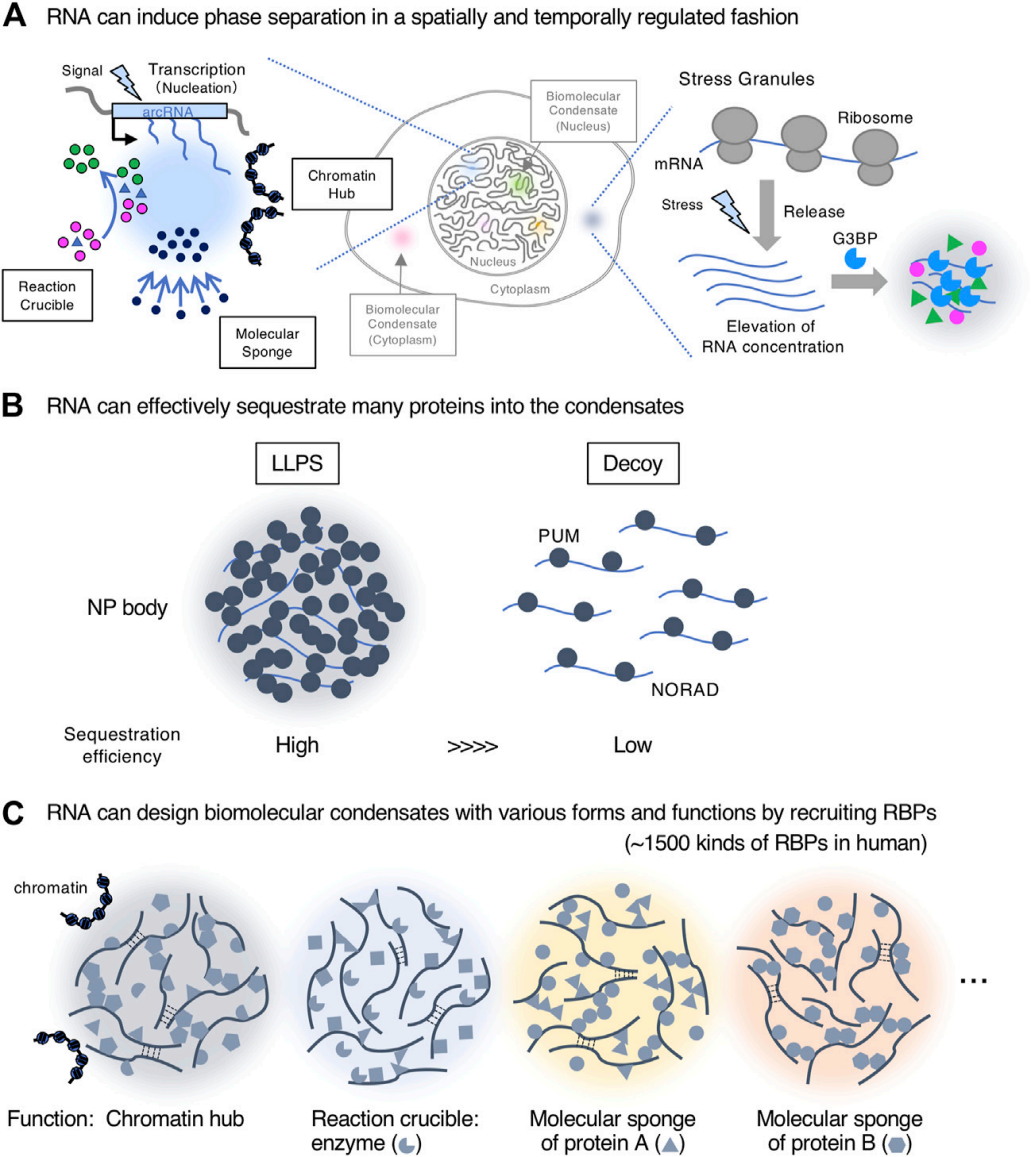
RNAs are suitable biomolecules for scaffolds of biomolecular condensates. **(A)**. RNA can induce phase separation in a spatially and temporally regulated manner. Transcription of nuclear architectural RNAs (arcRNAs) (nucleation event) induces nuclear condensates with roles such as reaction crucible, molecular sponge, and chromatin hub. The stress granule is shown as an example of the formation of cytoplasmic condensates with RNA scaffolds. **(B)**. RNA can effectively sequester many proteins into the condensates by liquid-liquid phase separation (LLPS) compared with a stoichiometric decoy mechanism. The NP (NORAD-PUM) body is shown as an example. **(C)**. RNA can create biomolecular condensates with various forms and functions by recruiting a wide variety of RNA-binding proteins (RBPs) (∼1,500 kinds of RBPs in humans).

We discuss three reasons why RNAs are suitable for scaffolding biomolecular condensates, with reference to recent findings [also see (Chujo et al., 2016; Yamazaki et al., 2019)] (Figure 2).

First, RNA can induce phase separation in a spatially and temporally regulated manner, enabling the local and transient control of nuclear processes, such as gene regulation and chromatin organization at a specific genomic position near the transcription site (Figure 2A). As cellular signals induce transcription of such RNAs, this mechanism would be useful for responses to environmental changes including cellular stressors. This idea is supported by how most arcRNAs, such as NEAT1_2, HSATIII (highly repetitive satellite III), and IGS (ribosomal intergenic spacer) lncRNAs, are stress-inducible (Chujo et al., 2016; Yamazaki, 2018). In the cytoplasm, RNAs can also induce temporally regulated phase separation. G3BP proteins recognize RNAs released from ribosomes under stress, and these interactions trigger phase separation to form stress granules (Guillén-Boixet et al., 2020; Yang et al., 2020) (Figure 2A). In addition, RNA copies produced by transcription, a distinct feature from DNA, increase the local concentration of arcRNAs (nucleation) that eventually induce phase separation. Condensates reportedly quickly disappear when transcription of arcRNAs is stopped (Fox et al., 2005; Mao et al., 2011; Shevtsov and Dundr, 2011). When degradation of arcRNAs is inhibited, the condensates become larger (Imamura et al., 2018; Fukushima et al., 2020; Machitani et al., 2020; Tanu et al., 2021). These data suggest that the continuous supply of RNAs by ongoing transcription and the RNA degradation rate can maintain the phase-separated state and temporal regulation of condensate formation. Although many lncRNAs are often expressed at low levels, such expression patterns can have a significant impact on the confined space via the phase separation mechanism if the valency of interaction among the lncRNAs and other molecules, such as RBPs, is high. Thus, lowly expressed lncRNAs may impact the biological processes at specific times and space via phase separation.

Second, RNA can effectively sequester many RBPs into the condensates through their short sequences, usually 4–17 nucleotides, or secondary structures (Lunde et al., 2007; Prikryl et al., 2011). This sequestration significantly impacts the freely available pool of RBPs. A recent study has shown that NORAD lncRNAs sequester Pumilio proteins (PUM) into NORAD-PUM (NP) bodies in the cytoplasm (Elguindy and Mendell, 2021). Quantitative analyses have revealed that NORAD efficiently sequesters 42-fold PUM through LLPS, involving NORAD-PUM and PUM-PUM interactions, compared with a stoichiometric decoy mechanism (Figure 2B). These data demonstrate the importance of phase separation in sequestration and how a smaller number of RNAs can impact the regulation of many proteins via this process.

Third, RNA can design biomolecular condensates with various forms and functions by recruiting a wide variety of RBPs through its sequence and structure. RNA is negatively charged, soluble, flexible, and usually a much longer polymer than protein, therefore making it suitable to act as a scaffold. More than 1,500 human RBPs, which possess a wide variety of biological functions, can bind RNAs. Consequently, RNAs can integrate the functions of these RBPs by assembling a variety of RBPs and forming the condensates. In the case of lncRNAs, as they do not need to have translatable open reading frames, their sequences may be only constrained by the requirements to design the structure and function of condensates (Figure 2C). Furthermore, RNA can scaffold various condensates with a specific shape, size, and structure by micellization like paraspeckles (described below) (Yamazaki et al., 2021).

From these features, lncRNAs (and protein-coding RNAs with non-coding functions) perform distinct functions that are not achieved by other biomolecules.

## NEAT1_2 lncRNA has multiple functional RNA domains that dictate form and function of the paraspeckle

One representative arcRNA is NEAT1_2 lncRNA, a scaffold of paraspeckle nuclear bodies localized adjacent to nuclear speckles. NEAT1_2 is a long intronless transcript (22.7 kb in humans) produced from the *NEAT1* gene by RNA polymerase II-mediated transcription and is essential for paraspeckle assembly (Chen and Carmichael, 2009; Clemson et al., 2009; Sasaki et al., 2009; Sunwoo et al., 2009; Naganuma et al., 2012). The *NEAT1* gene also encodes a short isoform NEAT1_1, which is dispensable for paraspeckle formation but has several important functions (Li R. et al., 2017; Naveed et al., 2020). The paraspeckle contains more than 60 protein components (Fox et al., 2002; Naganuma et al., 2012; Fong et al., 2013; West et al., 2014; Kawaguchi et al., 2015; Yamazaki and Hirose, 2015; Mannen et al., 2016; Fox et al., 2018; An et al., 2019; Barra et al., 2020; Chen et al., 2020; McCluggage and Fox, 2021). A recent study using a new method called HyPro-seq has expanded the paraspeckle proteins (PSPs) (Yap et al., 2022). Among the PSPs, several are essential for paraspeckle formation (Naganuma et al., 2012; Kawaguchi et al., 2015; Yamazaki et al., 2018). These PSPs are involved in NEAT1_2 stability, isoform switching, and NEAT1_2 RNP assembly (Naganuma et al., 2012; Kawaguchi et al., 2015; Yamazaki et al., 2018). Prion-like domains of FUS and RBM14, and NOPS and the coiled-coil domain of NONO are essential for paraspeckle assembly (Hennig et al., 2015; Yamazaki et al., 2018). In addition to the protein components, specific RNAs, including inverted Alu repeat-containing RNAs, CTN-RNA (cationic amino acid transporter two RNA), AG-rich RNAs, mRNAs of nuclear-encoded mitochondrial proteins, and poorly processed RNAs, are recruited to the paraspeckles (Chen and Carmichael, 2009; Clemson et al., 2009; Hu et al., 2015; West et al., 2016; Wang et al., 2018; Yap et al., 2022). In this manner, the paraspeckles sequester a particular set of proteins and RNAs to control gene expression (Chen and Carmichael, 2009; Clemson et al., 2009; Hirose et al., 2014; Imamura et al., 2014; Wang et al., 2018; Tanu et al., 2021). The paraspeckles can also interact with chromatin genome-wide that are enriched in active promoters and enhancer elements (West et al., 2014; Li X. et al., 2017; Sridhar et al., 2017; Fang et al., 2019; Bonetti et al., 2020; Cai et al., 2020). NEAT1_2 is induced by various stressors and pathological conditions, such as proteasome inhibition, viral and microbial infections, neurodegenerative diseases including amyotrophic lateral sclerosis (ALS) and frontotemporal dementia (FTD), fibrosis, and cancer/p53 activation (Nishimoto et al., 2013; Hirose et al., 2014) (Tollervey et al., 2011; Tsuiji et al., 2013; Imamura et al., 2014; Adriaens et al., 2016; Idogawa et al., 2017; Imamura et al., 2018; Fukushima et al., 2020; Rheinbay et al., 2020). These data suggest the general importance of NEAT1 in the stress response (McCluggage and Fox, 2021).

**FIGURE 3:**
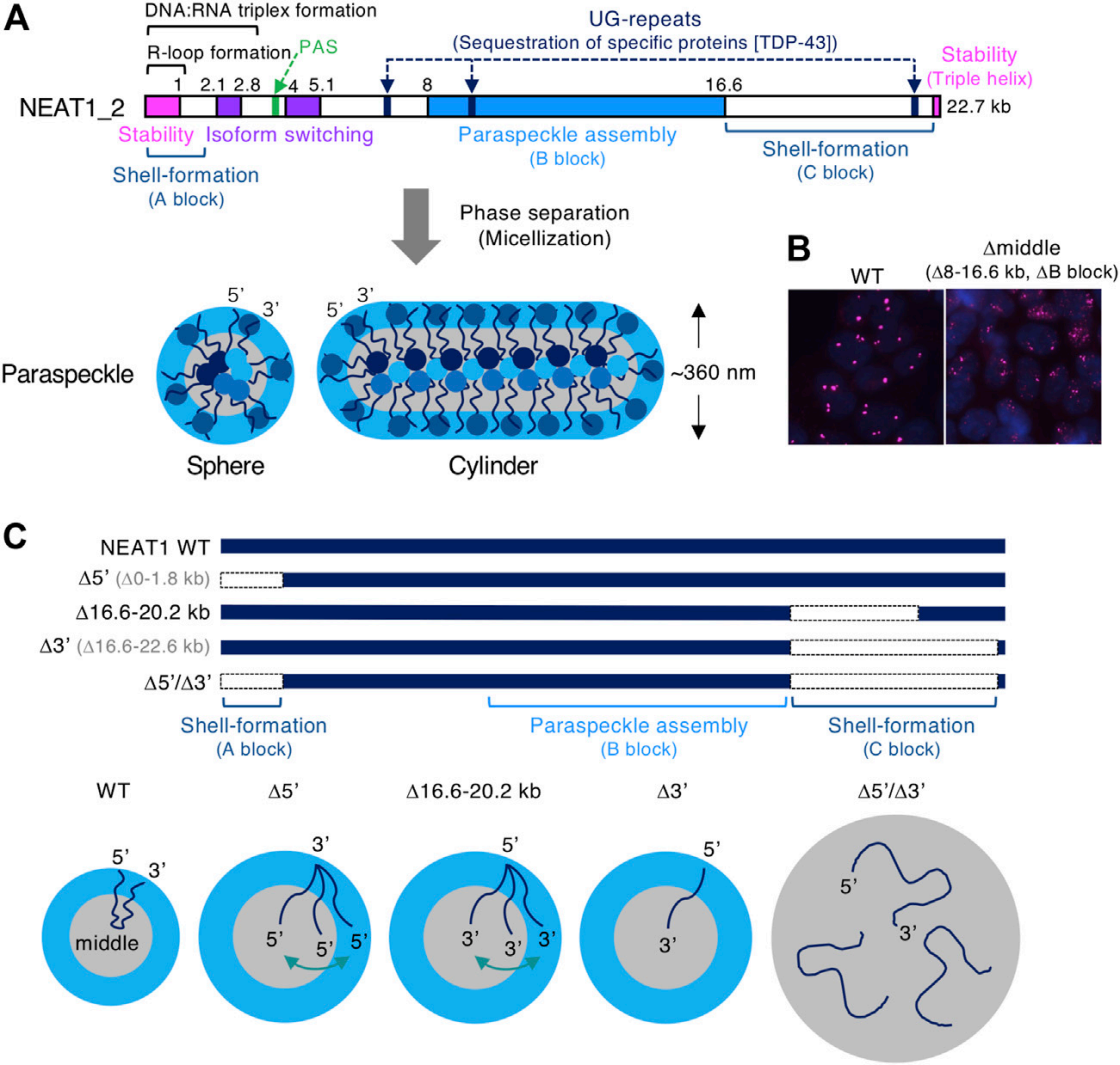
The functional RNA domains of human NEAT1 long non-coding RNA (lncRNA). **(A)**. Schematics show the domains of human NEAT1_2 lncRNA required for the form and function of paraspeckles. These domains include NEAT1_2 stability, isoform switching from NEAT1_1 to NEAT1_2, polyadenylation signal (PAS), UG-repeats that sequester TDP-43 proteins, R-loop formation (Dumelie and Jaffrey, 2017), DNA:RNA triplex formation (Sentürk Cetin et al., 2019), paraspeckle assembly (B block), shell-formation (A and C blocks). The spherical and cylindrical paraspeckles with restricted size (Sx: ∼360 nm in HeLa cells) form through micellization, a type of phase separation. **(B)** Deletion of the NEAT1_2 middle domain (8–16.6 kb region, B block) causes the formation of smaller paraspeckle foci (magenta). Nuclei are stained with DAPI (blue). **(C)** Schematics show NEAT1 mutants lacking the 5′ and/or 3′ domains (shell-forming domains) and the paraspeckles constructed by these mutants. Localization of NEAT1_2 within these paraspeckles and their size are shown.

NEAT1_2 lncRNA possesses multiple functional NEAT1 RNA domains required for stability, including a triple helix structure, isoform switching from NEAT1_2 to NEAT1_1, paraspeckle assembly, and recruitment of specific proteins (Brown et al., 2012; Wilusz et al., 2012; Yamazaki et al., 2018; Hirose et al., 2019; Yamazaki et al., 2019; Modic et al., 2019; Yamazaki et al., 2021) (Figure 3A). The major assembly domain is located on the NEAT1_2 middle domain (8–16.6 kb region of NEAT1_2), which is essential for constructing intact paraspeckles (Yamazaki et al., 2018) (Figure 3B). This NEAT1_2 middle domain increases the local concentration of paraspeckle core proteins, such as NONO and FUS, by recruiting these proteins to the RNA domain (Yamazaki et al., 2018). This feature shows the importance of the multivalent interaction in phase separation. Long UG repeats in NEAT1_2, which are evolutionally conserved in humans and mice, are essential for recruiting TDP-43 proteins that strongly bind UG stretches (Modic et al., 2019; Tollervey et al., 2011; Yamazaki et al., 2019) (Figure 3A). Accordingly, NEAT1_2 lncRNA domains determine the features of the paraspeckle.

## Micellization of RNP block copolymers: A newly identified mechanism in the formation of biomolecular condensates

The paraspeckle has several structural features: 1) highly organized internal core-shell structure, where 5′ and 3′ terminal regions (5′ and 3′ regions) of NEAT1_2 localize in the shell and the middle region localize in the core (PSPs are also localized in shell, core, and patch), 2) the paraspeckle shows a spherical or cylindrical shape with restricted short axes (Sx), 3) the paraspeckles are elongated to form cylindrical shapes by NEAT1_2 transcriptional upregulation (Souquere et al., 2010; Hirose et al., 2014; West et al., 2016; Yamazaki et al., 2018) (Figure 3A). We have recently identified the NEAT1 RNA domains for these features. The 5′ and 3′ RNA domains of NEAT1_2 determine the shell localizations of the 5′ and 3′ regions of NEAT1_2, respectively (Yamazaki et al., 2021). Deletion of either the 5′ or 3′ region causes the redistribution of either end into the core of the paraspeckle (Figures 3A,C). Furthermore, simultaneous deletion of both the 5′ and 3’ regions causes random distribution within the paraspeckles (Figures 3A,C). By applying soft matter physics theory to explain these features, we found that the paraspeckles form through micellization, a new intracellular phase separation mechanism of biomolecular condensates (Yamamoto et al., 2020b; Yamazaki et al., 2021). This model treats NEAT1_2 RNPs as block copolymers and paraspeckles as polymer micelles.

### Block copolymer and micellization

**FIGURE 4:**
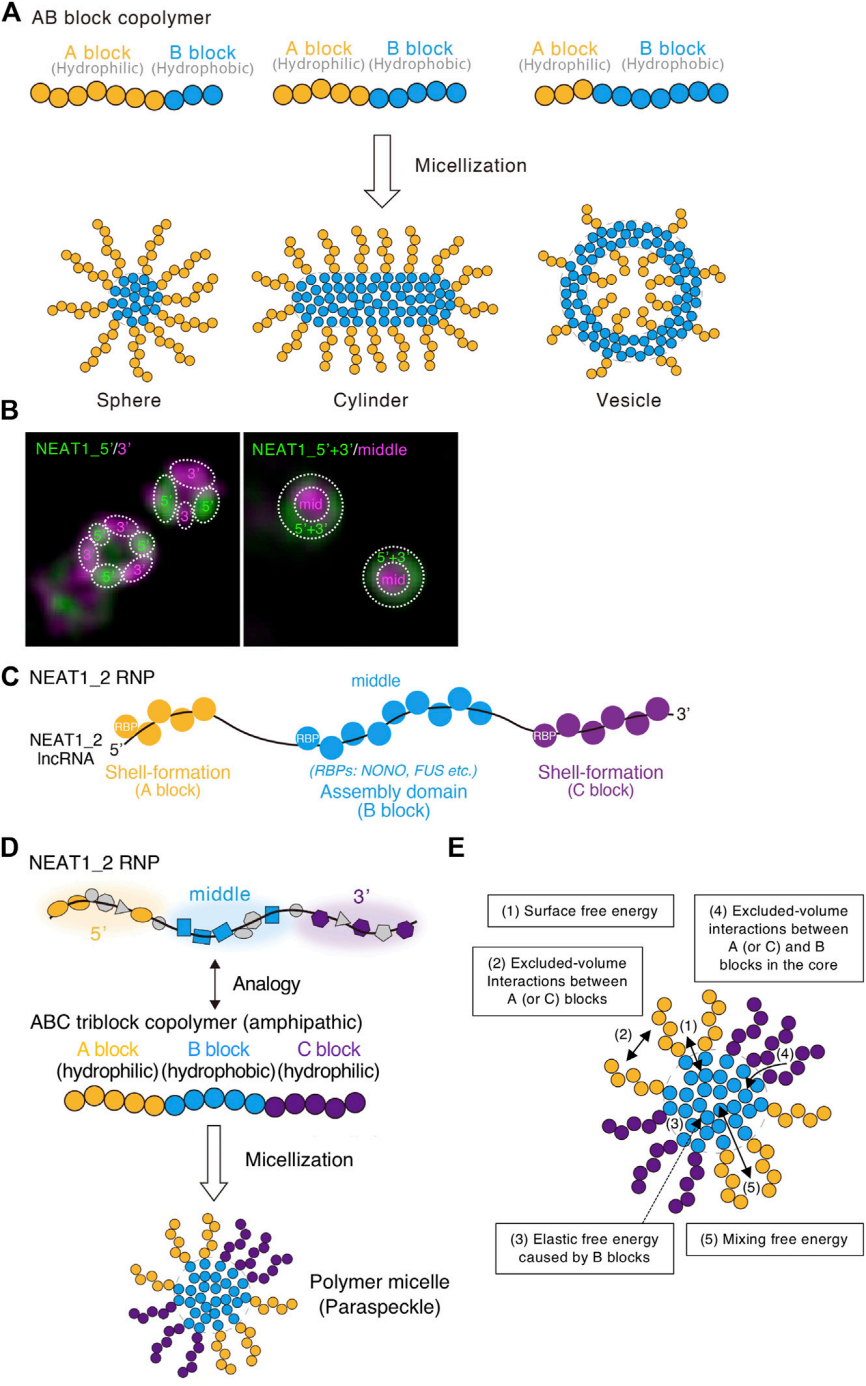
ABC triblock copolymer micelle model of the paraspeckle. **(A)** Schematics of AB amphipathic (di)block copolymers with different block lengths and the micelles they form in water. **(B)** The 5′ and 3′ domains of NEAT1_2 localize in distinct shell domains of the paraspeckle. The super-resolution images (structured illumination microscopy) with indicated probes are shown. Dotted circles indicate the domains within the paraspeckles where the 5′, 3′, and/or middle domains of NEAT1_2 localize. **(C)** RNA-binding proteins (RBPs) coat the shell-formation domains and the assembly domain of the NEAT1_2 long non-coding RNA (lncRNA). **(D)** ABC triblock copolymer micelle model of the paraspeckle. **(E)** Energetic contributions (1–5) considered in the ABC triblock copolymer micelle model of the paraspeckle are shown in a schematic.

The block copolymer consists of two or more chemically different polymers joined by covalent bonds. When there are two or three polymer blocks, the block copolymer is referred to as an AB block copolymer or ABC triblock copolymer, respectively. For an AB block copolymer, if the A block is hydrophilic and the B block is hydrophobic, then the AB block copolymer can form micelles by self-assembly of the B blocks in the core and localizing the A block on the shell in water. The micelles have various shapes, including spherical, cylindrical, lamellar, and vesicular, and are analogous to amphiphiles, such as detergents or phospholipids (Figure 4A). These internal organizations and shapes are similar to paraspeckles. The lengths of the polymer blocks determine the size of the micelles. Furthermore, the permutation of blocks along the copolymer determines the structure of the micelle and the configuration of the block copolymers within micelles (Moughton et al., 2012). The ratio of the A to B blocks mainly determines the shape (Mai and Eisenberg, 2012; Bates and Bates, 2017) (Figure 4A). As the fraction of the A block is reduced (and the fraction of the B block increases), the shape of the assembly changes from spherical, to cylindrical, lamellar, and vesicular (Figure 4A). The polymer concentration also contributes to determining the shape. This type of phase separation in which assemblies have optimal size, internal morphology, and shape is called micellization.

### ABC triblock copolymer micelle model of the paraspeckle

The NEAT1_2 middle domain is a major paraspeckle assembly domain, and the 5′ and 3′ domains are shell-formation domains (Yamazaki et al., 2018; Yamazaki et al., 2021) (Figure 3A). The NEAT1_2 5′ and 3′ regions localize in distinct areas in the shell of the paraspeckle (Figure 4B). As RBPs usually coat RNAs, we presume that RBPs bound to the 5′ and 3′ domains determine the hydrophilicity of the shells of the paraspeckle. The middle assembly domain of NEAT1_2 interacts with several oligomer-forming proteins such as NONO, which oligomerizes mainly through hydrophobic interactions (Passon et al., 2012; Yamazaki et al., 2018) (Figure 4C). Therefore, we treated NEAT1_2 RNPs as amphipathic ABC triblock copolymers, where the middle major assembly domain corresponds to the hydrophobic B block and the 5′ and 3’ domains correspond to the hydrophilic A and C blocks, respectively (Figure 4D).

To construct this block copolymer micelle model, we consider five free energetic contributions (Yamamoto et al., 2020b; Yamazaki et al., 2021) (Figure 4E):

1) The surface free energy of the core (B block) of the polymer micelle (the paraspeckle): this is the energy cost because the B block units at the surface of the core have fewer B block units at the neighbor to interact than the B block units in the interior of the core.
2) The free energy from the excluded-volume interactions between the A blocks and those between the C blocks in the shell: these interactions are repulsive because these blocks are hydrophilic and thus tend to mix with water.
3) The elastic free energy of B blocks: this is caused by the stretching of the B blocks (Doi, 1996). Because of their connectivity, polymers behave as (thermal) springs and favor shrunken states. Because A and/or C blocks are localized at the shell, B blocks are more stretched as the size of the paraspeckle core is enlarged (Semenov, 1985). A previous report estimates an approximately 20-fold compaction of NEAT1_2 within the paraspeckle (Souquere et al., 2010).
4) The free energy from the excluded-volume interactions between the A and B blocks or between the C and B blocks: these are free energy costs because entering the A (or C) blocks (the shell) into the B blocks (the core) disturbs the interaction between the B block units.
5) The mixing free energy from thermal fluctuations: this free energy decreases when A or C blocks between the core and the shell are randomly distributed.

Free energetic contributions (1, 2, and 3 in Figure 4E) can explain the size of wild-type (WT) paraspeckles because both the 5′ and 3’ domains localize in the shell and the middle domain localizes in the core. This structure does not change with the upregulation of transcription. When the size of the paraspeckle becomes large, the surface free energy of the core (1 in Figure 4E) decreases, whereas the free energy from the repulsive interactions between the A blocks or C blocks (2 in Figure 4E) and the elastic free energy (3 in Figure 4E) becomes large (Semenov, 1985; Halperin and Alexander 1989; Zhulina et al., 2005). The system evolves to decrease the free energy while nascent NEAT1_2 is added as its transcription proceeds. The interaction free energy and elastic free energy make micellization different from LLPS. If there is no repulsive interaction, condensates grow without bound as long as components are available. This is indeed the case with condensates formed by LLPS. These repulsive interactions and elastic free energy limit the incorporation of NEAT1_2 into the assemblies, influencing the size and number of the assemblies.

To consider the internal morphology changes observed in the NEAT1_2 mutants, we consider the energetic contributions shown as 4 and 5 in Figure 4E. These free energies influence the balance of energetic contributions shown as 1, 2, and 3 in Figure 4E. Therefore, the balance of the energies (1–5) defines the size, shape, and internal morphology of the assemblies. As a result, the block copolymer micelle has optimal size, shape, and internal morphology.

Our model also considers the transcription dynamics of NEAT1_2. Thus, we can predict how the transcription rate influences the size and internal morphology of the paraspeckle (Yamamoto et al., 2020b).

### Experimental validation of the ABC block copolymer model of the paraspeckle

Our ABC triblock copolymer micelle model can explain features of the paraspeckle constructed by WT and mutant NEAT1_2.

#### A and C blocks are essential for the core-shell internal architecture of the paraspeckle

Our micelle model can explain the organization of the NEAT1_2 ends within the paraspeckles in NEAT1_2 mutants. The model predicts that as the A or C block becomes shorter, the A or C block is redistributed to the core of the paraspeckle (Yamazaki et al., 2021). Consistent with this prediction, partial deletion of the NEAT1_2 3′ region (16.6–20.2 kb) (the C block) results in a random distribution of the 3′ region. Further deletion of the NEAT1_2 3′ region (16.6–22.6 kb) results in complete redistribution of the 3′ region into the core (Yamazaki et al., 2021) (Figure 3C). In addition, NEAT1_2 lacking the 5′ and 3′ domains (Δ5’/Δ3’) (the A and C blocks) forms paraspeckles without internal ordered structures (Yamazaki et al., 2021) (Figure 3C).

#### A and C blocks limit the size (short axis) and number of paraspeckles by restricting the incorporation of NEAT1_2 molecules into the paraspeckle

Our micelle model predicts that the length of the A or C block determines the number of NEAT1_2 molecules in a paraspeckle and the size/Sx. Deletion of the A (or C) block increases the number of NEAT1_2 molecules in a paraspeckle by reducing the repulsive excluded-volume interactions between the hydrophilic A (or C) blocks. Size/Sx is limited by excluded-volume interactions between the A (and C) blocks (and the elastic free energy of the B blocks: discussed in the following section) (Figure 3C).

**FIGURE 5:**
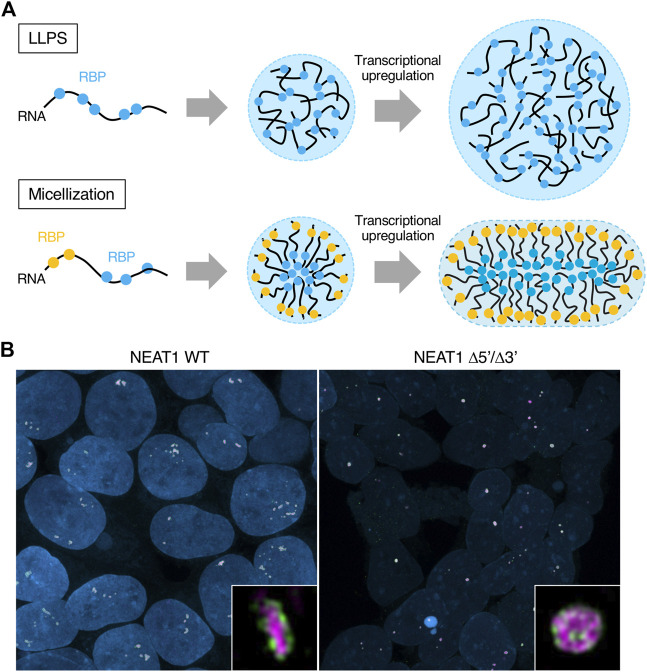
Differences of the condensates formed by liquid-liquid phase separation LLPS and micellization. **(A)** Schematics show condensates formed by LLPS and micellization. Their internal morphologies and behaviors upon transcriptional upregulation are also shown. Different RNA-binding proteins (RBPs) are illustrated as other color circles (blue and yellow). The condensates formed by LLPS and micellization can contain many types of RBPs, although the illustration shows one or two types of RBPs for simplicity. **(B)** Representative images of the paraspeckles in HAP1 NEAT1 wild type (WT) and Δ5’/Δ3′ cell lines are shown. Insets are magnified images of the paraspeckles in these cell lines. The 5′ domains of NEAT1_2 are shown in green and the middle domains of NEAT1_2 are shown in magenta. Nuclei are shown in blue.

Experimentally, the paraspeckles become large when deleting the 5′ and/or 3′ domains. The extreme case is the NEAT1_2 Δ5’/Δ3′ mutant (Figure 3C). This mutant lacks most of the hydrophilic domains (A and C blocks), reducing or losing repulsive interactions between NEAT1_2 RNPs. In this case, condensates form likely through LLPS. Paraspeckles constructed by the NEAT1_2 Δ5’/Δ3′ are ∼2-fold larger in average Sx than WT paraspeckles (Yamazaki et al., 2021). We estimated the number of NEAT1 lncRNA molecules per paraspeckle in the NEAT1 Δ5′, Δ3′, and Δ5’/Δ3′ mutants compared with WT. This estimation showed a ∼2-fold increase in the Δ5′ and Δ3′ mutants and ∼3-fold increase in the Δ5’/Δ3′ mutant (Yamazaki et al., 2021). As a spherical WT paraspeckle contains about 50 NEAT1_2 lncRNAs (Chujo et al., 2017), a paraspeckle in these NEAT1 mutants contains 100–150 NEAT1_2 lncRNAs (Chujo et al., 2017; Yamazaki et al., 2021). Taken together, the presence of the 5′ and 3’ domains of NEAT1_2 switches the paraspeckle formation process from LLPS to micellization (Figures 5A,B).

The B block is the hydrophobic core of the condensates, where the NONO proteins form oligomers mainly through hydrophobic interactions (Passon et al., 2012). When the length of the B block is constant, the lengths of the A and C blocks determine the size of the condensate. As the length of the B block becomes shorter, the size of the condensate becomes smaller. As described above, deleting the NEAT1_2 5′ and 3’ domains (the A and C blocks) increased the Sx of the paraspeckle. Deletion of the NEAT1_2 assembly domain (the B block) (e.g., major assembly domain, the middle domain [8–16.6 kb]) formed much smaller paraspeckles than WT paraspeckles (Yamazaki et al., 2018) (Figure 3B). Thus, the balance of the length of A/C blocks and the B block determines the condensate size.

Paraspeckles are reportedly often found as clusters containing multiple paraspeckles (Visa et al., 1993; Hirose et al., 2014; Yamazaki et al., 2021) (Figure 5B, left). Block copolymer micelles rarely coalesce each other. When the sizes of the micelles are smaller than their optimal sizes, they can coalesce. However, the coalescence kinetics are much slower than the fusion of LLPS-condensates because their micelle shells become barriers to contacts between the cores of the micelles. When NEAT1_2 expression levels are constant and the number of NEAT1_2 molecules in a paraspeckle increases, the number of paraspeckles is reduced. Because the number of NEAT1_2 lncRNAs per paraspeckle increases in the NEAT1 Δ5′, Δ3′, and Δ5’/Δ3′ mutants, there are fewer paraspeckles in these mutants (Yamazaki et al., 2021). In particular, most of the paraspeckles in the NEAT1 Δ5’/Δ3’ mutant form paraspeckles as a single entity, likely through LLPS (Yamazaki et al., 2021) (Figures 5A,B).

#### Excluded-volume interactions between A and C blocks and elastic free energy of the B block determine the shape of the paraspeckle

**FIGURE 6:**
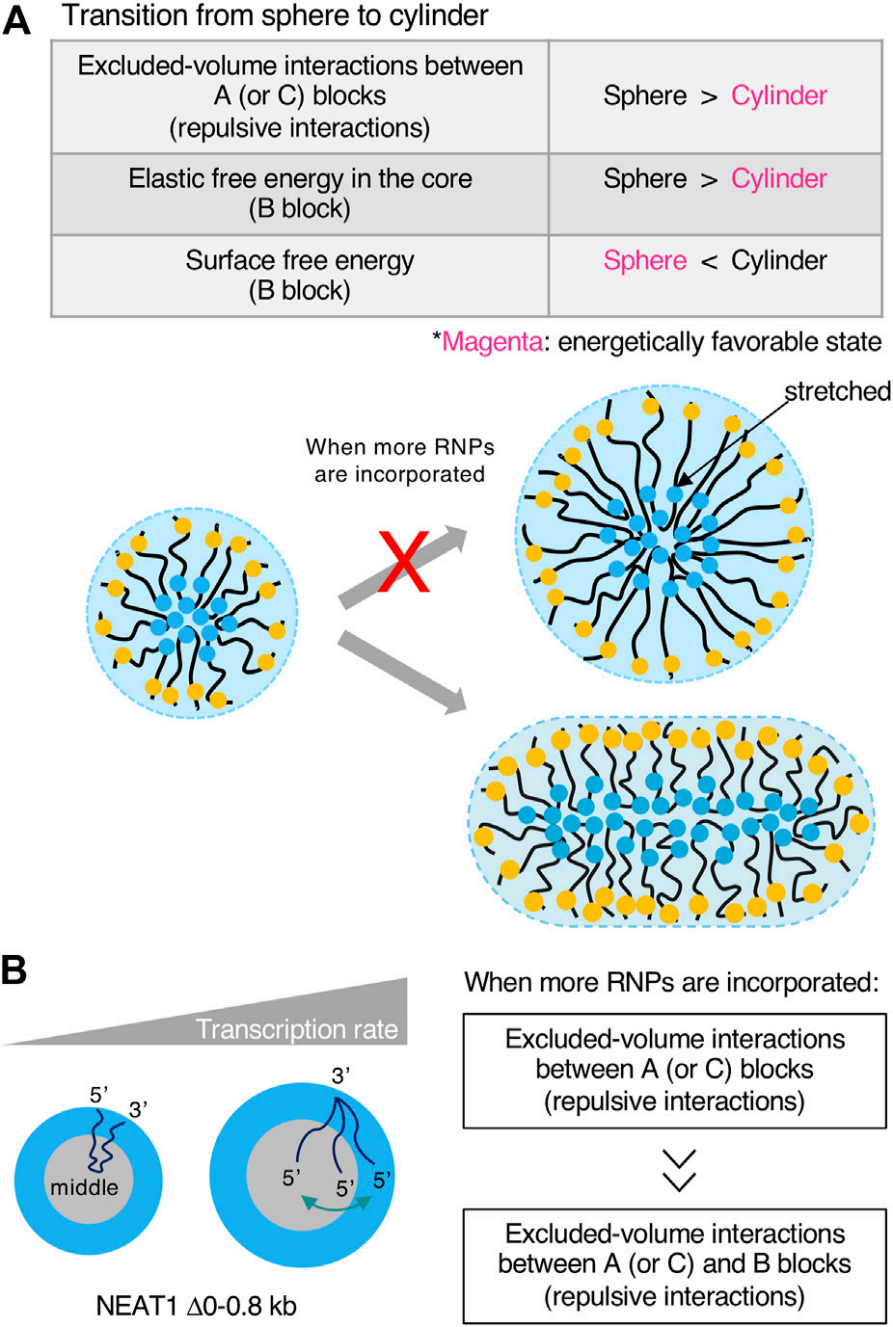
Transcription rates influence the shape and internal morphology of the condensates. **(A)** Free energetic contributions in the sphere-cylinder transition are shown in a table. Free energetically favored states (shape) are shown in magenta when more RNA-protein complexes (RNPs) are incorporated into condensates. Schematics of the transition are also shown. If a large micelle forms, the shape is energetically unfavorable from elastic free energy caused by stretches of RNP polymers in the core. **(B)** The contribution of the transcription rate in the internal morphology of condensates is shown. Excluded-volume interactions between A (or C) blocks become dominant compared with excluded-volume interactions between A (or C) and B blocks in the core when more RNPs are incorporated into the condensates upon transcriptional upregulation.

Our micelle model can explain the shape of the paraspeckle. The WT paraspeckles show cylindrical as well as spherical shapes, while the NEAT1_2 Δ5’/Δ3’ mutant forms large spherical paraspeckles likely through LLPS, in which the condensates become spherical by minimizing the surface free energy (Figure 5B). The transition of the shape from a sphere to a cylinder is determined by the competition among surface free energy of the B blocks, the repulsive interactions between the A blocks and between the C blocks, and the elastic free energy of the B block in the core (Yamazaki et al., 2021) (Figure 6A). As the condensates become larger, the contribution of the elastic free energy of the B block becomes dominant. Then, the shape changes from a sphere to a cylinder, which has a shorter Sx than a sphere. This reduces the elastic free energy of the B blocks and the excluded-volume interactions between the A or C blocks.

#### The transcription rate determines the size and internal morphology of the paraspeckle

Our micelle model predicts how the production of NEAT1_2 by transcription influences the Sx of the paraspeckle. As the transcription rate increases, the number of NEAT1_2 molecules associated with the paraspeckle increases (Yamazaki et al., 2021). The number of NEAT1_2 molecules in a paraspeckle is determined by the competition between the stabilizing and destabilizing factors. The stabilizing factor is the multivalent interactions between the middle domain (B block) of NEAT1_2. The destabilizing factors are the excluded-volume interactions between terminal blocks in the shell and the elastic energy from the stretching of the middle domain (Yamazaki et al., 2021) (Figure 3C). In contrast to the micellization, paraspeckles constructed by the NEAT1_2 Δ5’/Δ3’ mutant become larger as NEAT1_2 expression levels increase (Yamazaki et al., 2021) (Figures 5A,B).

Our model also predicts that the transcription rate influences the internal morphology of the paraspeckle. As the transcription rate increases, A (or C) blocks tend to relocalize to the B block (the core) (Figure 6B). As the production of NEAT1_2 increases, the repulsive interactions between A blocks and between B blocks dominate the repulsive interactions between A (or C) blocks and the B blocks in the core (Yamazaki et al., 2021) (Figure 6B). In our experiment, when NEAT1_2 expression levels increased, the fraction of the shell decreased in the NEAT1 mutant lacking the 5’ domain (Yamazaki et al., 2021).

The paraspeckle has relatively constant Sx because of the size limitation in the micellization (Yamazaki et al., 2021). Lack of A and C blocks (hydrophilic blocks that generate repulsive interactions) causes high variability in the size of the paraspeckles, as observed in the NEAT1 Δ5’/Δ3’ mutant paraspeckles (Yamazaki et al., 2021) (Figure 5B). Transcription may be a key determinant of this size variability. The duration and/or strength of NEAT1_2 transcription bursts may influence the size and shape of the paraspeckle. Furthermore, the frequency of the bursts may influence the number of paraspeckles. This hypothesis is supported by a previous study which demonstrated that NEAT1 transcriptional activation by mitochondrial signals can increase the number of elongated paraspeckles (Wang et al., 2018).

As described in this article, we identified a new intracellular phase separation mechanism using theoretical analyses. We termed block copolymers made of RNPs as “RNP block copolymers”. Therefore, a method combining molecular biology experiments and theoretical physics is a powerful approach to investigate the mechanism of intracellular phase separation.

## Potential functional importance of micellization

There are several differences between LLPS (macroscopic phase separation) and micellization. As described above, in LLPS, the condensates typically have a spherical shape and show coarsening and coalescence to minimize the surface free energy. However, micellization has several features, including 1) optimal size, 2) optimal shape (such as sphere, cylinder, lamellar, vesicle), 3) optimal internal morphology, and 4) rare coalescence of the condensates. These features determined by micellization would be related to functions of the condensates. Here, we discuss the potential functional importance of these features of the paraspeckle, which may generalize the significance of the condensates formed by micellization in cells.

### Generating the core-shell architecture of condensates

**FIGURE 7:**
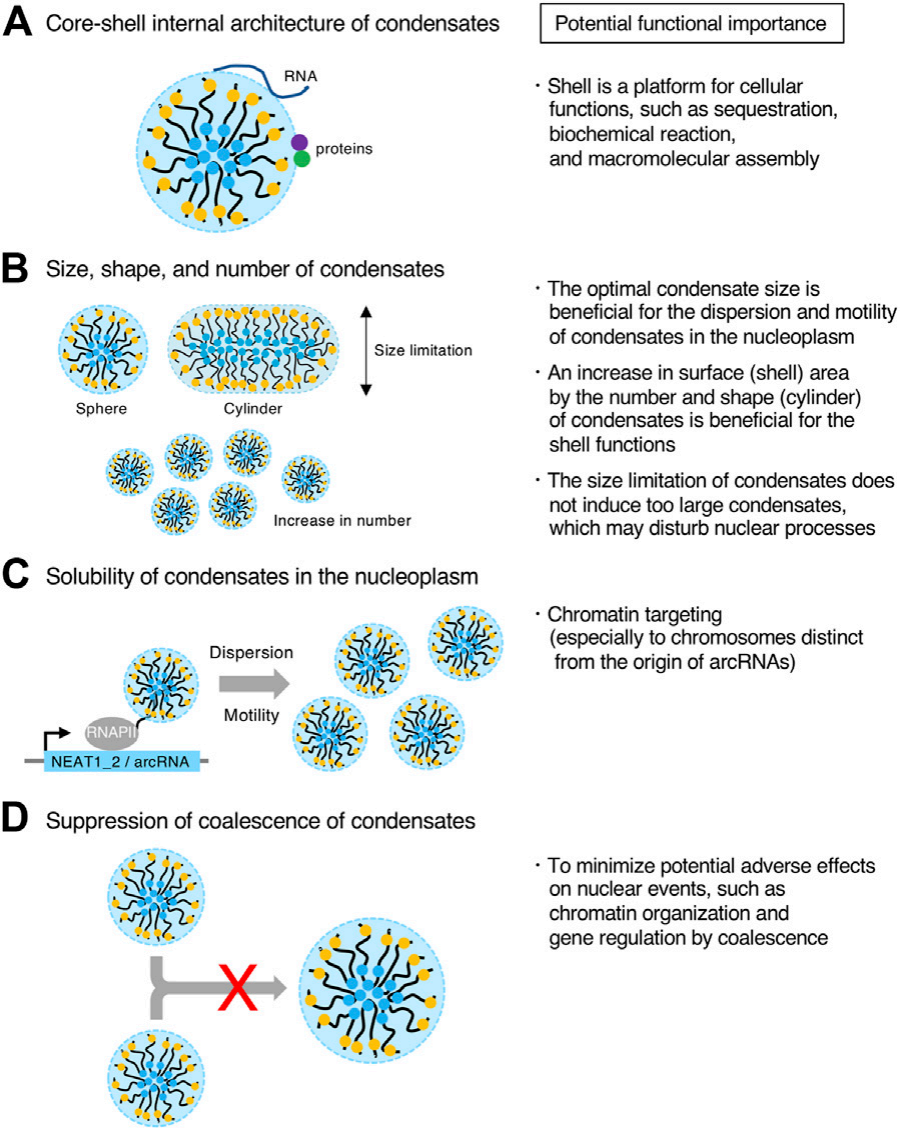
Potential functional importance of micellization. **(A–D)** Features of condensates formed through micellization are shown (Left). Potential functional importance related to the features on the left is listed (Right).

A distinct feature of the paraspeckle is the core-shell internal architecture, which is evolutionally conserved (Souquere et al., 2010; Cornelis et al., 2016; West et al., 2016; Yamada et al., 2022). As various proteins and RNAs are sequestered at the paraspeckles, the shell may be a platform to gather specific proteins and RNAs, as described above (Figure 7A). For example, the AG-rich RNAs are sequestered at the shell of the paraspeckles, suggesting the importance of the shell for this process (West et al., 2016). In block copolymer micelles, the shells are thought to be sparse. In the paraspeckle, the electron density of the shell of the paraspeckle is low (Souquere et al., 2010). Thus, the shell may act as a nest for interacting factors of NEAT1_2 RNPs to perform sequestration, biochemical reactions, and macromolecular assembly (Figure 7A).

### Controlling the size, shape, and number of condensates

LLPS condensates grow without bound by coarsening and coalescence as long as the components are available. By contrast, in micellization, the size of the condensate is restricted by the length of the block copolymer. Thus, this micellization mechanism will be useful for tighter size control of condensates compared with other size control mechanisms, such as the elastic energy of the cytoskeleton, kinetic limitations on coarsening, stoichiometric constraints, multiple nucleation sites, stabilization of condensate surface by RNAs, nascent RNAs as surfactants, microemulsion, and emulsification including the Pickering stabilization (Brangwynne et al., 2009; Berry et al., 2018; Style et al., 2018; Garcia-Jove Navarro et al., 2019; Dar and Pappu, 2020; Martin et al., 2020; Ranganathan and Shakhnovich, 2020; Rosowski et al., 2020; Sabari et al., 2020; Wei et al., 2020; Folkmann et al., 2021; Hilbert et al., 2021; Snead and Gladfelter, 2021; Yamamoto et al., 2021; Mittag and Pappu, 2022). NEAT1_2 forms spherical or cylindrical paraspeckles with relatively constant Sx (approximately 360 nm on average Sx in HeLa cell line) from the size limitation of micellization (Souquere et al., 2010; Yamazaki et al., 2021). In contrast, NEAT1 Δ5’/Δ3’ mutant paraspeckles form larger condensates than WT ones. In this way, WT paraspeckles do not become too large. Thus, the paraspeckles may have optimal sizes to perform their functions (Figure 7B). For example, it may be possible that condensates that are too large have difficulties diffusing in the nucleoplasm, possibly by the interference of nuclear structures (Figure 7B). In addition, large condensates in the nucleus could disturb nuclear processes, such as chromatin organization and gene expression. Thus, this size limitation might minimize such adverse effects (Figure 7B).

The paraspeckles often have cylindrical shapes, and this feature is evolutionally conserved in humans, mice, and opossums. This shape is possibly important for its functions. A cylinder has a larger surface area than a sphere, which may be important for the shell functions such as sequestration, as described above (Figure 7B). In addition, cylindrical paraspeckles are reportedly less dynamic than spherical paraspeckles and have increased capability of mRNA sequestration (Wang et al., 2018). Further studies will be required to understand the molecular mechanism and functional importance of the cylindrical shape of the paraspeckle.

The number of NEAT1_2 RNPs that are incorporated into a paraspeckle is limited by repulsive interactions between the RNPs. When the NEAT1_2 lncRNA expression levels are the same, the number of condensates is larger in micellization than in LLPS. This increased number of condensates, which also increases the condensate surface area, would be beneficial for efficient sequestration and widespread chromatin interactions (Figure 7B).

### Making condensates soluble in the nucleoplasm

It has been reported that paraspeckles that form near the *NEAT1* gene locus are released from the locus to the nucleoplasm (Mao et al., 2011; Shevtsov and Dundr, 2011). In the NEAT1 Δ5’/Δ3’ mutant, the paraspeckles form a few large spherical condensates per nucleus, presumably localized near the NEAT1_2 transcription sites (Yamazaki et al., 2021) (Figure 5B). Therefore, the hydrophilic surface of the micellar structure (solubility), as well as the size of condensates, may be important for dispersion or motility of paraspeckles in the nucleoplasm by solubilization (Figure 7C). This dispersion could contribute to genome-wide targeting of the paraspeckles to chromatin (West et al., 2014; Li X. et al., 2017; Sridhar et al., 2017; Bonetti et al., 2020; Cai et al., 2020).

### Suppressing coalescence of condensates

During micellization, coalescence of the assemblies is suppressed. This feature may be necessary to minimize adverse effects from coalescence (Figure 7D). If paraspeckles frequently coalesce, this may influence various nuclear events such as chromatin organization and gene expression because the NEAT1_2 lncRNAs are highly expressed, frequently interact with chromatin, and function in regulating gene expression. A recent report using the CasDrop system has shown that the fusion of condensates formed by LLPS reorganizes the nuclear chromatin architecture and connects chromatin (Shin et al., 2018). Instead, diffusion of the condensates may be functionally important during micellization, as described above.

As discussed here, these characteristics and structures are likely related to the functions. Further investigations will help reveal the functional importance of the micellization of RNP block copolymers.

## Future perspectives

We have investigated the paraspeckle as a model to understand how RNAs can form biomolecular condensates. From these investigations, we propose a new concept that RNPs can act as block copolymer micelles and form micelles in cells. An additional question requiring further investigation is what the molecular determinants of the RNP block copolymer micelles are. As RBPs usually coat RNAs, we reason that some proteins interacting with the shell-forming domains contribute to shell formation of the RNP block copolymer micelles (Figure 4B). Indeed, we recently identified proteins that contribute to shell formation (T. Yamazaki, unpublished observation). Thus, it would be important to understand how these RBPs function. In addition to analyzing these RBPs, it would be important to elucidate the specific RNA sequences and structures to construct RNP block copolymers by mainly determining the interacting RBPs. Unlike synthetic block copolymers, the binding sites of the RBPs that determine A (hydrophilic) or B (hydrophobic) blocks may be not uniform in the RNAs. The ratio and number of these RBPs would determine the domain as an A (hydrophilic) or B (hydrophobic) block. It is tempting to speculate that the RBPs determining the A block (hydrophilic) would be required for the dispersion of RNPs, such as messenger RNPs that seem to not form condensates, which might be beneficial for their biogenesis. Furthermore, it would be essential to use constructive approaches to understand requirements for the formation of RNP block copolymer micelles and the determinants of their functionality. These analyses will elucidate the molecular basis of how RNPs act as block copolymers and open the door to create designer condensates using RNPs with various structures, physical properties, and functions, according to the design guidelines of block copolymer micelles, called “designer soft materials”.

Currently, the molecular mechanisms and theoretical principles of some structural features of the paraspeckles remain unknown. These features include how 1) the NEAT1_2 RNPs are bundled, 2) the NEAT1_2 5′ and 3’ domains occupy distinct shell domains, and 3) the paraspeckle proteins localize in the patch, as well as the shell and core (Kawaguchi et al., 2015; West et al., 2016). Further work, including dissection of the NEAT1 RNA domains and theoretical analyses, would help answer these questions.

Another critical question is how the micelles eventually play physiological and pathological roles through their molecular functions. As described, the micelles possibly contribute to the molecular functions, such as acting as a molecular sponge or chromatin hub. Various physiological roles of NEAT1 have been reported. NEAT1 is physiologically important in mammary gland development and lactation, corpus luteum formation, and the establishment of pregnancy (Nakagawa et al., 2014; Standaert et al., 2014). As described above, NEAT1 is relevant to various diseases including cancer, viral infection, ALS, and FTD. Thus, NEAT1 would be an interesting model to understand the links among features of RNP block copolymer micelles, molecular functions, and physiological and pathological roles.

Finally, it is essential to understand how widely micellization is used in biological systems. For example, the nuclear stress body, a primate-specific, stress-induced nuclear condensate constructed by HSATIII lncRNA, has a sea-island structure, a typical structure formed by block copolymers (Chiodi et al., 2000; Kawaguchi et al., 2015). It may be also conceivable that if biomolecular condensates are small, then the condensates formed by micellization may not be distinguishable from those formed by LLPS because of microscope resolution limitations. Thus, micellization may be overlooked and widely used.
